# PROTOCOL: Effects of preconception care and periconception interventions on maternal nutritional status and birth outcomes in low‐ and middle‐income countries: A systematic review

**DOI:** 10.1002/cl2.1007

**Published:** 2019-07-13

**Authors:** Zohra S. Lassi, Sophie G.E. Kedzior, Jai K. Das, Zulfiqar A. Bhutta

**Affiliations:** ^1^ Robinson Research Institute, Adelaide Medical School University of Adelaide Adelaide South Australia Australia; ^2^ Department of Pediatrics Aga Khan University Karachi Sindh Pakistan; ^3^ Global Child Health The Hospital for Sick Children Toronto Ontario Canada

## 1 BACKGROUND

### 1.1 The problem, condition or issue

Interest in preconception health for maximising gains for mothers and babies started with the release of the seminal report from Centre for Disease Control (Johnson et al., 2006). Further, in 2011, the World Health Organisation (WHO) convened a meeting of experts where there was an overwhelming agreement on the potential for preconception care to have a positive impact on maternal and child health outcomes (World Health Organisation, 2013). Since then there is growing awareness of the importance of the preconception period and efforts have been made to increase awareness and promote reproductive health from adolescents onwards.

Preconception care is important for healthy maternal, birth, and neonatal health outcomes (Dean et al., 2013). Optimising a woman's health before planning and conceiving pregnancy is increasingly recognised as an important strategy to enhance maternal and child health (Dean, Imam, Lassi and Bhutta, 2013). Preconception period is an ideal time to introduce interventions relating to nutrition and other lifestyle factors to promote health and for ensuring good pregnancy preparedness. Since 99% of all maternal and newborn deaths occur in low‐ and middle‐income countries (LMICs; World Health Organisation, 2017), early start of preconception care particularly for girls living in LMICs is very crucial. At present, policies and guidelines on preconception care are scarce and the care starts when the women becomes pregnant and extends to childbirth and postnatal period (for mothers and babies). There is a clear gap in the continuum of care, particularly for young girls who enters the reproductive years and women who are not pregnant. These girls and women receives little to no attention until their first pregnancy. Evidence also suggests that antenatal care is often too late to revert the detrimental health risks and issues that may have on developing foetus (Dean et al., 2013).

Adolescents face multiple challenges to their health and social well being if they become pregnant early in life. Approximately 13% of all maternal mortality occurs in adolescents (World Health Organisation, 2014). The risk of maternal mortality is approximately five times higher for adolescents under the age of 15 years and twice as high for adolescents between 15 and 19 years of age compared with women aged 20–29 years (Nove, Matthews, Neal, & Camacho, 2014). These girls are at a higher risk for developing hypertension during pregnancy, severe anaemia, bleeding and infection. Because their pelvis has not yet grown large enough for the baby to pass through the birth canal, hence they have higher risk of obstructed labour, stillbirths; and their newborns are also more likely to be born prematurely, have low birth weight, or die in the first month of life (Gibbs, Wendt, Peters, & Hogue, 2012; Paranjothy, Broughton, Adappa, & Fone, 2009; World Health Organisation, 2007). These risks are further exacerbated by factors such as poverty, illiteracy, limited access to health care, lack of social support from family and absence of autonomy for decision‐making (Nove et al., 2014). Apart from direct health consequences to the mother and baby, early motherhood is often linked to school drop‐out, social difficulties and poor socioeconomic status (Penman‐Aguilar, Carter, Snead, & Kourtis, 2013). They also have higher odds of experiencing depressive symptoms including loneliness, sleep disorders, loss of appetite and even thoughts of harming oneself or the baby within the 3 months after birth (Reid & Meadows‐Oliver, 2007). The evidence further details that children born to teenage mothers tend to have poor health, poor cognitive development, behavioural problems and poor educational outcomes; they also have a high probability of becoming a teen parent themselves (Black et al., 2002). Therefore, it is important to encourage the use of contraceptives and educate the importance of planning pregnancy and delaying first pregnancy until the woman is at least 18 years of age which allows a woman's body to fully mature.

Maternal nutritional deficiencies particularly iron and folate are common in LMICs. Anaemia in women from LMICs is due to low dietary intake of bioavailable iron combined with endemic infectious diseases such as helminthiasis, which puts women at increased risk during pregnancy. Low preconception haemoglobin and ferritin levels increase the risk of poor foetal growth and low birth weight (Dean, Lassi, Imam, & Bhutta, 2014a). Similarly, folate deficiency can lead to the development of neural tube defects (NTDs) in the foetus. Other micronutrients such as zinc, vitamin B and calcium have been found to improve maternal and newborn outcomes when supplementation is provided during pregnancy; however, their impact during the preconception period has not been established (Ramakrishnan, Grant, Goldenberg, Zongrone, & Martorell, 2012). Improved reproductive health and planning is the fundamental component of preconception care and starting early interventions such essential nutritional supplements in the preconception period can help women begin her pregnancy in the best of her health.

### 1.2 The intervention

Each year an estimated 140 million births take place (World Health Organisation, 2018). Of these 16 million occur to adolescents between the ages of 15–19 years and approximately 2.5 million to girls less than 16 years of age (World Health 2018, 2018). It is therefore important to delay the age at first pregnancy and optimise the interpregnancy intervals and make a healthy start when pregnancy is planned by supplementing essential micronutrients.

Many adverse maternal, neonatal and pregnancy outcomes may be avoidable if the age at first pregnancy or optimising the gap between two pregnancies. Previous evidence has shown the benefits of delayed childbearing, specifically in adolescence, as adolescent pregnancy is known to be associated with an increased risk of preterm birth, stillbirth, small‐for‐gestational age, neonatal mortality, and complications during labour and delivery (Haldre, Rahu, Karro, & Rahu, 2007; Paranjothy et al., 2009; World Health Organisation, 2007). There is, however, variable evidence related to prolonging interpregnancy intervals. In a systematic review, Conde‐Agudelo, Rosas‐Bermudez, Castaño, and Norton (2012) identified that compared with interpregnancy intervals of 18–23 months, interpregnancy intervals shorter than 6 months were associated with increased risks of preterm birth, low birth weight and small‐for‐gestational age babies. While delaying the age of first pregnancy ensures the maturation and growth of the body, optimising pregnancy intervals gives time for body to recover and prepare itself for another pregnancy. This review will consider interventions to delay the age of first pregnancy or to optimise birth intervals. Interventions such as health education, contraception education and distribution, individual counselling, or sex education, and so forth. may be population‐based, community‐based, school based, hospital/clinic based targeting specific groups such as teenage or delivered by health professionals or workers.

On the other hand, the benefits of micronutrient supplementation during pregnancy are well‐established, particularly for iron and folic acid. There are numerous nutrition related interventions targeting different vitamins and nutrients to improve maternal and neonatal outcomes. Whilst folic acid may be one of the most widely known, there is evidence that multivitamins and other nutrients have a critical role in brain and nervous system development as well as impact the immune system during pregnancy, specifically relating to the inflammatory response (Ramakrishnan et al., 2012). Specifically, interventions have shown that vitamin A received during pregnancy may reduce maternal anaemia in women who likely have a vitamin A deficiency, however this review also demonstrated the vitamin A did not reduce maternal or newborn mortality (McCauley, van den Broek, Dou, & Othman, 2015). Another review investigating the use of supplementing pregnant women with vitamin D, demonstrated that vitamin D may reduce the risk of preeclampsia, low birth weight, and preterm birth (De‐Regil, Palacios, Lombardo, & Peña‐Rosas, 2016). There is also evidence for the use of multivitamin supplementation with iron and folic acid to reduce the risk of miscarriage (Balogun et al., 2016).

However, there is limited evidence for micronutrient supplementation specifically during pre‐ and periconception apart from the use of folic acid, which has shown to reduce NTD (De‐Regil, Peña‐Rosas, Fernández‐Gaxiola, & Rayco‐Solon, 2015). The intermittent utilisation of iron and folic acid prior to conception has shown to reduce the risk of anaemia in reproductive age women, though additional evidence is needed to support improvements in other maternal and newborn outcomes (Fernández‐Gaxiola & De‐Regil, 2011). Ideally many of these interventions would have a preventive focus, for example, iron and folic acid supplementation, food fortification or dietary diversification to decrease the incidence of anaemia in women before they become pregnant.

### 1.3 How the intervention might work

Pregnancy in the teenage years is associated with multiple risks and delaying the age of first pregnancy can reduce these risks. Interventions such as sex education and counselling at school and at community settings by peers and community health workers have shown impact (Brieger, Delano, Lane, Oladepo, & Oyedrian, 2001; García et al., 2012). Such interventions improve knowledge and promote attitudinal and behaviour change among young adolescents. These interventions also promote use of condoms and other birth control mechanism including abstinence (Cabezón et al., 2005). Interventions to delay pregnancy can include health education, contraception education and distribution, skills building and different forms of counselling (Oringanje et al., 2009). One intervention commonly utilised in LMICs is cash transfer programmes to encourage adolescent women to stay in school for longer and as a consequence avoid early marriage or sexual initiation (Baird, Chirwa, McIntosh, & Özler, 2010). A systematic review by Khan, Hazra, Kant, and Ali (2016) assessed the evidence for using conditional and unconditional cash transfers as a method to encourage contraceptive use in LMICs. The majority of the included studies utilised cash transfers to encourage school attendance or aimed to improve overall health and nutrition. While there were few available studies specifically targeting contraception, some studies did demonstrate a positive impact on contraceptive use and a decrease in fertility outcomes (i.e., number of pregnancies resulting in live births).

Similarly, optimising the birth interval has shown positive impacts for mothers and babies (Afeworki, Smits, Tolboom, & van der Ven, 2015). Studies have long shown that interpregnancy intervals of <12 months or >60 months have an adverse effect on perinatal outcomes such as preterm birth, low birth weight, small for gestational age babies and congenital defects in babies (Dean, Lassi, Imam, & Bhutta, 2014b). While short pregnancy intervals <12 months are associated with anaemia, puerperal endometritis, premature rupture of membrane, longer intervals >60 months are associated with preeclampsia, third trimester bleeding and foetal death (Dean et al., 2014b). Furthermore the risks for folate and other nutritional deficiencies, cervical insufficiency, suboptimal breastfeeding, incomplete healing of uterine scar from previous caesarean delivery, and abnormal remodelling of endometrial blood vessels are higher for short interval and closely‐spaced pregnancies (Conde‐Agudelo et al., 2012). Therefore, it is important to intervene to delay the age of first pregnancy and optimise the intervals between the two pregnancies. There are numerous approaches that an intervention to promote birth spacing for women of reproductive age may undertake. Strategies may involve policies or population‐based interventions, or a combination of school and community‐based approaches (Aslam et al., 2015). Much like interventions focusing on delaying pregnancy, strategies that encourage women and couples to employ suitable spacing between births may involve health education, skills building and contraception education and distribution to ensure appropriate and consistent use of contraceptives (Aslam et al., 2015; Dean et al., 2014b). As per Aslam et al. (2015), these interventions may work by encouraging mothers to pursue educational avenues or work related accomplishments, in order to develop self‐confidence, self‐esteem and autonomy. A recent review on birth spacing interventions in low‐, middle‐, and high‐income countries found studies with high quality evidence for a positive impact for repeat pregnancy/birth (Norton, Chandra‐Mouli, & Lane, 2017). Successful interventions included those that targeted adolescents to teach them planning skills, including activities that involve preparing contraceptive plans.

Folate plays an important role in protein synthesis and metabolism and other processes related to cell multiplication and tissue growth. Its deficiency during pregnancy causes megaloblastic anaemia and accumulates homocysteine in the serum which is associated with an increased risk in cardiovascular disease, late pregnancy complications such as preeclampsia, and NTDs around the time of conception (Lassi & Bhutta, 2012). The literature shows that iron supplementation during pregnancy can be a protective factor against low birth weight, and given alone or with folic acid it is effective in increasing iron stores and preventing anaemia during later gestation (Fernández‐Gaxiola & De‐Regil, 2011). Since women might not know when they become pregnant, it is therefore important to ensure iron and folic acid sufficiency from early life. Health promotion campaigns are a common strategy to increase a population's knowledge and awareness on the benefits and importance of nutritional supplementation. Two systematic reviews (Chivu, Tulchinsky, Soares‐Weiser, Braunstein, & Brezis, 2008; Rofail, Colligs, Abetz, Lindemann, & Maguire, 2012) found that while these campaigns were successful in increasing overall awareness, knowledge, and consumption of folic acid before and during pregnancy, the increase in folic acid consumption was nowhere near optimal despite women having significantly increased knowledge. Interventions specific to adolescent nutrition are often conducted in school‐based settings, one meta‐analysis conducted by Salam et al. (2016) demonstrated that school‐based delivery significantly reduced anaemia.

### 1.4 Why it is important to do the review

While there is increasing evidence to support the provision of preconception care, the effectiveness of interventions to delay the age of first pregnancy, optimise birth intervals, and to provide periconception iron and folic acid supplement require further investigation. Determining the most effective delivery mechanisms across different settings is vital to successfully implementing pre‐ and peri‐conception interventions in LMIC settings (Poels, Koster, Boeije, Franx, & van Stel, 2016). While there are existing reviews examining the effects of various interventions on preventing teen pregnancies (Dean et al., 2014b; Oringanje et al., 2009), they have mainly included randomised controlled trials (RCTs). In this review, we will also include evidence from large‐scale programme evaluations in addition to relevant experimental studies, as randomisation is not always possible for all settings and populations. We want to ensure that our review is comprehensive and includes nonrandomised studies as contextual and supplementary evidence for the included RCTs (Schünemann et al., 2013). These studies can benefit systematic reviews by demonstrating whether an intervention works in different populations, whether there are possible interaction effects and can describe long‐term outcomes.

We did not identify any review of interventions to optimise pregnancy intervals. Existing reviews have only examined pregnancy intervals from observational studies (Brown, Allen, & Torkelson, 2013; Conde‐Agudelo et al., 2012; Dean et al., 2014b), therefore it is important to review the evidence of interventions to prolong interpregnancy intervals and their impact on maternal nutrition and birth outcomes.

Existing reviews have also evaluated the effects of periconception folic acid use (Dean et al., 2014a; De‐Regil et al., 2015; Ramakrishnan et al., 2012) and iron‐folic acid use (Fernández‐Gaxiola & De‐Regil, 2011) but these reviews are outdated, therefore, there is a need to update the evidence from RCTs and other large scale programme evaluation.

This review aims to synthesise the evidence on the effectiveness of preconception care interventions relating to delayed age at first pregnancy, optimising interpregnancy intervals, periconception folic acid, and periconception iron‐folic acid supplementation on maternal, pregnancy, birth, and child outcomes by systematically reviewing the primary studies, along with rigorous evaluations of existing programmes. This approach will enable a comprehensive assessment of the effectiveness of these interventions for improving maternal, birth and child health and nutrition outcomes. For example, maternal outcomes for this review include pregnancy, anaemia, use of birth control methods, knowledge and attitudes about the risk of unattended pregnancies and maternal mortality. Birth and child health outcomes include perinatal/neonatal mortality, the presence of neural tube defects and pre‐term birth. For this review nutritional outcomes can be related to anaemia as well measurements relating to folate such as red blood cell folate or serum folate. This evidence will be critical to inform policy and programmatic decision‐making in LMICs.

## 2 OBJECTIVES

The overall objective is to assess the effectiveness of the following pre and periconception interventions when compared with no/standard intervention on maternal nutrition, birth and neonatal outcomes in LMICs.

1. Interventions to delay age at first pregnancy.
2. Interventions to optimise interpregnancy intervals on maternal nutrition, birth and neonatal outcomes.
3. Periconception folic acid supplementation.
4. Periconception iron‐folic acid supplementation.

## 3 METHODOLOGY

### 3.1 Criteria for including and excluding studies

#### 3.1.1 Types of study designs

We will include primary studies, including large‐scale programme evaluations, to assess the efficacy and/or effectiveness of interventions using the following study designs:

- RCTs, where participants were randomly assigned, individually or in clusters, to intervention and comparison groups. Cross‐over designs will be eligible for inclusion.
- Quasi‐experimental designs, which include:
  (a) Natural experiments: studies where nonrandom assignment is determined by factors that are out of the control of the investigator. One common type includes allocation based on exogenous geographical variation.
  (b) Controlled before‐after studies, in which measures were taken of an experimental group and a comparable control group both before and after the intervention. We also require that appropriate methods were used to control for confounding, such as statistical matching (e.g., propensity score matching, or covariate matching) or regression adjustment (e.g., difference‐in‐differences, instrumental variables).
  (c) Regression discontinuity designs; here, allocation to intervention/control is based upon a cut‐off score.
  (d) Interrupted time series (ITS) studies, in which outcomes were measured in the intervention group at least three time points before the intervention and after the intervention.

Pre‐post studies without a control group will not be included.

#### 3.1.2 Types of participants

The target population is women of reproductive age* (i.e., 10–49 years) including adolescent girls, regardless of health status, living in LMICs. Country income will be classified according to the 2018 World Bank list of country economies (World Bank, 2017). We will compare the World Bank list of country economies over years and consider all those studies conducted in countries which were part of LMICs before 2018. Interventions are aimed at nonpregnant women, but that the outcome measurement is aimed at pregnant women and their children. For optimising birth intervals, we will consider interventions given while they are pregnant to optimise birth intervals for the next pregnancy.

#### 3.1.3 Types of interventions

The following interventions targeting women of reproductive age (10–49 years) including adolescent girls (10–19 years) during the pre‐ and peri‐conception period in LMICs will be included:

- Interventions to delay age at first pregnancy such as curriculum based sex education, abstinence alone programmes, interactive computer based interventions, and so forth.
  a. Educational interventions and contraceptive promotion provided at the community, school or household level by parents, colleagues, teachers, health workers, or social workers to adolescents and young women
- Interventions to optimise interpregnancy intervals such as introducing family planning methods, abstinence alone programmes, and so forth.
  a. Educational interventions and contraceptive promotion provided at the community, school or household level by parents, colleagues, teachers, health workers or social workers to mothers of reproductive age.
- Periconception folic acid
  a. Pubescent or menstruating women who received any folic acid supplementation before conception and continued using until the first trimester of pregnancy.
- Periconception iron‐folic acid
  a. Pubescent or menstruating women who received any iron‐folic acid supplementation before conception and/or continued using until the first trimester of pregnancy.

These interventions will be compared against no intervention, standard of care (whatever is applicable in the setting the study was conducted) or placebo. Folic acid and iron‐folic acid use only during pregnancy will not be included. We will exclude multiple micronutrient powders for point‐of‐use fortification of foods, fortification of staple foods, water, condiments or seasonings with folic acid or iron and other micronutrients or the provision of oral contraceptives that contain folic acid in this review. We will exclude fortification programmes because they are employed universally and no exact period of start and end of intake would be known to generate evidence for recommendation. We will exclude oral contraceptives that contain folic acid because they merit a separate review.

#### 3.1.4 Types of outcome measures

Primary outcomes

- Maternal
  a. Unintended pregnancy
  b. Anaemia
  c. Iron deficiency anaemia
- Neonatal
  a. Neural tube defects
  b. Stillbirth
  c. Perinatal mortality
  d. Neonatal mortality
  e. Low birth weight

Secondary outcomes

- Maternal
  a. Reported changes in knowledge and attitudes about the risk of unintended pregnancies
  b. Initiation of sexual intercourse
  c. Use of birth control methods
  d. Serum folate
  e. Adverse effects
  f. Adherence to folic acid or iron‐folic acid supplementation
  g. Abortion
  h. Miscarriage
  i. Maternal mortality
- Neonatal
  a. Preterm birth
  b. Small‐for‐gestational age
  c. Other congenital anomalies
  d. Admission to special care for any cause

Studies will not be excluded if the outcomes of interests are not reported. We will consider outcomes measured at any time points during pregnancy and the postpartum period. They will be pooled at a reasonable time point reported by most of the studies.

### 3.2 Duration of follow‐up

For folic acid and iron‐folic acid supplementation: We will consider studies if folic acid and iron‐folic acid are supplemented during both the pre‐ and peri‐conception period.

For interventions to delay the age at first pregnancy: we will consider studies that have provided intervention at anytime during preconception period.

For interventions to optimise interpregnancy intervals: we will consider studies that have provided interventions to optimise interpregnancy intervals at any time during the previous pregnancy or even after giving birth to the last child.

#### 3.2.1 Types of settings

Any setting in low and middle‐income countries.

## 4 SEARCH STRATEGY

We will not impose any restrictions, for example, language or publication status or the publication dates, on the literature searches described below.

### 4.1 Electronic searches

The search will be performed in the following electronic databases: CABI's Global Health, CINAHL,Cochrane Controlled Trials Register (CENTRAL), Dissertation Abstracts International, Embase, Epistemonikos, ERIC, HMIC (Health Management Information Consortium), MEDLINE, Popline, PsycINFO, Scopus, Social Science Index from Web of Science, Sociofiles, WHO's Global Health Library, WHO Reproductive Health Library, and the WHO nutrition databases (http://www.who.int/nutrition/databases/en/). We will also search the websites of selected development agencies or research firms (e.g., JOLIS, IDEAS, IFPRI, NBER, USAID, and World Bank) and Google Scholar.

The search strategy can be found in Appendix A.

## 5 SEARCHING OTHER RESOURCES

We will check reference lists of all included studies and systematic reviews for additional references. We will make every effort to contact relevant organisations and experts in the field to identify unpublished or ongoing studies. References of included articles, relevant reviews, and annotated bibliographies will be scanned for eligible studies.

## 6 DESCRIPTION OF METHODS USED IN PRIMARY RESEARCH

For this review, the primary research design of interest will be experimental and quasi‐experimental study designs as well as nonrandomized studies with a control group, including CBA. We will also accept ITS studies with at least three time points before and three time points after the intervention. The studies will have women in preconception period and periconceptional period in case of folic acid supplementation exposed to intervention to delay age at first pregnancy or optimise pregnancy intervals or supplementation of iron and folic acid. Studies will also have women in periconceptional period for the supplementation of folic acid. One of the representative study of interest for this review is Mba, Obi and Ozumba (2007). This is an intervention study to evaluate the impact of reproductive health education on the knowledge and attitude in rural Nigerian community. The study compared adolescents in a secondary school that received health education on reproductive health with another secondary school (control group) that did not receive.

## 7 CRITERIA FOR DETERMINATION OF INDEPENDENT FINDINGS

Before initiating the synthesis (detailed below), we will ensure that all articles reporting on the same study are appropriately linked. To ensure independence and appropriate combination of outcome constructs, syntheses will be conducted according to the type of interventions specified above. If multi‐arm studies are included, intervention groups will be combined or separated into different forest plots, and we will ensure that there is no double counting of participants. If an outcome is reported in several different metrics, we will perform unit conversions in order to pool the data. We do anticipate differences in the types of literature and we will ensure that any analysis will take possible sources of dependency into account by grouping papers into studies and ensuring that no double counting of evidence takes place when synthesising across studies.

## 8 DETAILS OF STUDY CODING CATEGORIES

Two review authors (Z. S. L. and S. K.) will extract data independently and a third review author (J. K. D.) will check for reliability and resolve any conflict. We will extract the primary data for the study characteristics including details of the populations, setting, sociodemographic characteristics, interventions, comparators, outcomes and study design in duplicate. We will check primary study data for accuracy. Disagreements will be resolved by discussion or consultation with a third reviewer.

The following information will be extracted for each included study:

- Background: time period when study took place, type of publication (e.g., full‐text journal article, abstract, conference paper, thesis), study country or countries, funding source(s) and conflicts of interest
- Population and setting: population age and setting
- Methods: Study design, description of study arms, unit of allocation, sample, or cluster size per study arm (for individually or cluster randomised trials respectively), start and end date, follow‐up
- Participants: total number randomised/allocated, sociodemographic data
- Intervention group details: number randomised/allocated to group, description of intervention, duration and follow‐up, timing, delivery of intervention, providers and their training. We will describe all the study intervention arms in the tables of included studies, however, we will only report the intervention arms that meet review inclusion criteria.
- Comparison group details: number randomised to group, description of comparison, duration and follow‐up, timing, providers and their training.
- Outcomes: measurement tool, validation of the tool, total number in intervention and comparison groups, and change indicated at each time point.

## 9 SELECTION OF STUDIES

Two review authors will independently screen titles and abstracts of all retrieved references. We will retrieve the full‐text study reports for all citations that at least one review author considers potentially relevant. Two review authors will independently screen the full text articles and identify studies for inclusion, as well as record reasons for exclusion of ineligible studies in a “Characteristics of excluded studies” table. We will resolve any disagreement through discussion or, if required, we will consult a third review author. We will identify and exclude duplicates and collate multiple reports of the same study so that each study, rather than each report, is the unit of interest in the review. We will record the selection process in sufficient detail to complete a Preferred Reporting Items for Systematic Reviews and Meta‐Analyses (PRISMA) flow diagram (Moher, Liberati, Tetzlaff, & Altman, 2009)

## 10 STATISTICAL PROCEDURES AND CONVENTIONS

We will use in RevMan to calculate the treatment effects (Review Manager, 2014). We will use risk ratio for dichotomous outcomes. We will use the mean difference for continuous outcomes reported on the same scale, and the standardised mean difference for continuous outcomes reporting the same outcome but measured on different scales in different studies included in the same meta‐analysis. We will express uncertainty with 95% confidence intervals for all effect estimates. When means and standard deviations are not reported, we will use other available data (e.g., confidence intervals, *T* values, *p* values) and appropriate methods described in the Cochrane Handbook for Systematic Reviews of Interventions (Higgins et al., 2011) to calculate the means and standard deviations.

## 11 TREATMENT OF QUALITATIVE RESEARCH

We do not plan to include qualitative research.

## FUNDING INFORMATION

Funding for this review came from a grant from the Bill & Melinda Gates Foundation to the Centre for Global Child Health at The Hospital for Sick Children (Grant No. OPP1137750)

## CONFLICT OF INTERESTS

The author declares that there are no conflict of interests.

## AUTHOR CONTRIBUTIONS

Please give a brief description of content and methodological expertise within the review team. It is recommended to have at least one person on the review team who has content expertise, at least one person who has methodological expertise and at least one person who has statistical expertise. It is also recommended to have one person with information retrieval expertise. Please note that this is the *recommended optimal* review team composition. Content: Z. L., S. K., J. D., Z. B. Systematic review methods: S. K., Z. L., J. D. Statistical analysis: Z. L. Information retrieval: S. K. and Z. L.

## APPENDIX A

### Search strategy

#### PubMed

(‘‘preconception care’’[MeSH] OR preconception[tiab] OR periconception[tiab] OR pre‐pregnancy[tiab] OR adolescent[MeSH] or adolescen*[tiab] OR OR Teen*[tiab] OR “prepregnancy”[tiab] OR “pre pregnancy”[tiab]) AND (“family planning”[tiab] OR “reproductive planning”[tiab] OR “contraception”[MeSH] or contracepti*[tiab] OR “birth control”[tiab] OR “contraception devices”[tiab] OR condom*[tiab] OR “intra uterine device”[tiab] OR “cervical cap”[tiab] OR pill OR sterilisation[tiab] OR “sexual abstinence”[tiab] OR “birth spac*”[tiab] OR “pregnancy interval”[tiab] OR “intergenesic interval”[tiab] OR “pregnancy spacing”[tiab] OR “interpregnancy interval”[tiab] OR delay[tiab] OR iron[MeSH] OR iron[tiab] OR "folic acid"[MeSH] OR "folic acid"[tiab])

#### Embase

(prepregnancy care/de OR preconception:ti,ab OR Periconception:ti,ab OR pre‐pregnancy:ti,ab OR adolescent/de or adolescent:ti,ab OR teenage:ti,ab OR Teen:ti,ab OR “prepregnancy”:ti,ab OR “pre pregnancy”:ti,ab) AND (“family planning”:ti,ab OR “reproductive planning”:ti,ab OR “contraception”/de or contraception:ti,ab OR “birth control”:ti,ab OR “contraception devices”:ti,ab OR condom:ti,ab OR “intra uterine device”:ti,ab OR “cervical cap”:ti,ab OR pill OR sterilisation:ti,ab OR “sexual abstinence”:ti,ab OR “birth spacing”:ti,ab OR “pregnancy interval”:ti,ab OR “intergenesic interval”:ti,ab OR “pregnancy spacing”:ti,ab OR “inter pregnancy interval”:ti,ab OR delay:ti,ab OR iron/de OR iron:ti,ab OR "folic acid"/de OR "folic acid":ti,ab)
